# Theories of Willpower Affect Sustained Learning

**DOI:** 10.1371/journal.pone.0038680

**Published:** 2012-06-22

**Authors:** Eric M. Miller, Gregory M. Walton, Carol S. Dweck, Veronika Job, Kali H. Trzesniewski, Samuel M. McClure

**Affiliations:** 1 Department of Psychology, Stanford University, Stanford, California, United States of America; 2 Department of Psychology, University of Zurich, Zurich, Switzerland; 3 Department of Psychology, University of California Davis, Davis, California, United States of America; University of California, Davis, United States of America

## Abstract

Building cognitive abilities often requires sustained engagement with effortful tasks. We demonstrate that beliefs about willpower–whether willpower is viewed as a limited or non-limited resource–impact sustained learning on a strenuous mental task. As predicted, beliefs about willpower did not affect accuracy or improvement during the initial phases of learning; however, participants who were led to view willpower as non-limited showed greater sustained learning over the full duration of the task. These findings highlight the interactive nature of motivational and cognitive processes: motivational factors can substantially affect people’s ability to recruit their cognitive resources to sustain learning over time.

## Introduction

Acquiring new cognitive abilities often requires sustained, effortful engagement with challenging tasks. Evidence suggests that even for the most talented individuals, becoming an expert in a new domain can require over 10,000 hours of training [1]. Because of these steep requirements, the ability to persist in cognitively demanding tasks is crucial for achievement in many fields. We demonstrate that beliefs about the nature of willpower can promote or hinder learning on a strenuous mental task that taxes working memory.

Recent findings have highlighted the pronounced role that implicit theories about the nature of intelligence and personality traits play in shaping behavior [2], [3]. Most relevant to the current study, implicit theories about willpower have been shown to moderate the extent to which self-control suffers following a demanding mental task [4]. Those participants who held, or were primed to hold, a “limited resource theory”–that is, who viewed, or were led to view, willpower as dependent on a resource that is easily be depleted through mental exertion–showed worse response inhibition and performance following a task with strong self-control demands. In contrast, holding the belief that mental exertion can be energizing (what we refer to as the “non-limited resource theory”) eliminated these deficits.

If the non-limited resource theory has a relative positive effect on people’s ability to sustain self-control, might these theories comparably affect cognitive growth in situations where learning requires sustained persistence on a strenuous task? We assess this by manipulating implicit theories about willpower and measuring sustained learning–improvement in performance (i.e., accuracy) over a series of trials on an extended, continuously challenging task that taxes working memory (see also [5]). Given the substantial attentional demands of this task, we predicted that participants primed to view willpower as relying on a limited resource would be less able to maintain the focus necessary to sustain learning compared to participants primed to view willpower as non-limited.

## Methods

Fifty-six college students were randomly assigned to the “limited” or “non-limited” willpower group. Written informed consent was obtained from all participants, and the study was approved by the Institutional Review Board at Stanford University. Following past research, implicit theories about willpower were manipulated through 8-item biased questionnaires intended to elicit agreement with one or the other theory about willpower [4] (see Text S1 for the full list of items contained in the questionnaires). For example, participants assigned to the limited resource theory group rated their agreement with items such as, “Working on a strenuous mental task can make you feel tired such that you need a break before accomplishing a new task.” Participants assigned to the non-limited resource theory group rated their agreement with items such as, “Sometimes, it is energizing to be fully absorbed with a demanding task.” Participants responded to each item on a 4-point scale with 4 indicating maximum agreement. In each group, participants expressed strong agreement with the items (Figure S1; *M*_limited_ = 3.27, and *M*_non-limited_ = 3.03; scale midpoint = 2.50), one-sample *t*s>6.80, *p*s<10^−7^).

The primary dependent measure was performance on a 20-minute (540 trial) spatial 3-back task. On each trial, an X appeared on the screen in one of four locations for 0.5 seconds. Participants were instructed to press one of four buttons, corresponding to the location of the stimulus that appeared three trials before the present trial. Successful performance required continuous updating and maintenance of working memory. Improvement in performance over time constituted our measure of sustained learning.

## Results and Discussion

We hypothesized that theories about willpower would influence participants’ ability to sustain learning over time. Thus, we predicted that the two groups would demonstrate comparable accuracy and improvement initially but that participants in the non-limited condition would show sustained improvement over the full task. To test these predictions, we examined the effect of limited vs. non-limited condition on a two-piece linear growth model (e.g., [6]). We used a two-piece model because we hypothesized that participants in both groups would show improved performance in the beginning of the task but over time the performance of participants in the limited condition would level off or drop whereas that of participants in the non-limited condition would continue to improve. We simplified the model by dividing the 540 trials into eight equal-size blocks and modeled growth across the first four blocks and the second four blocks using Mplus 6.1 (the findings did not differ when alternative numbers of blocks were tested; see Text S2 for further information regarding this analysis method).

**Figure 1:**
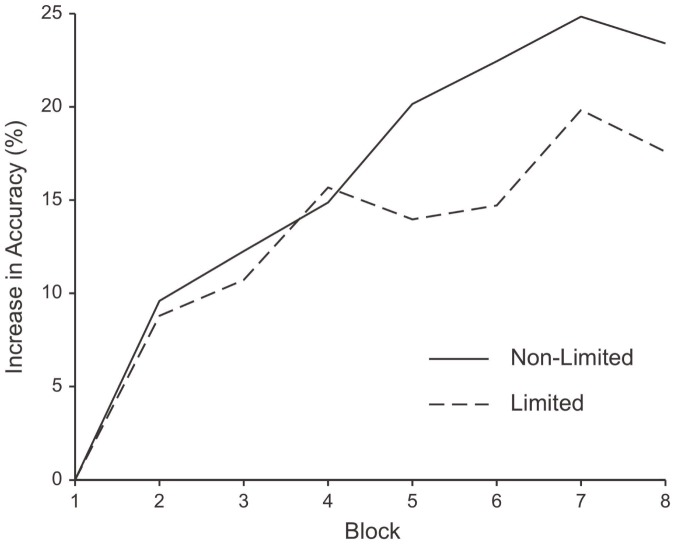
Learning over time on 3-back task. Improvement in percent accuracy on the 3-back task relative to baseline for the limited and non-limited willpower groups over the full 20-minute time course (each block is averaged over 67 trials).

We found no effect of condition on the intercept (b = .066, SE = .052, *p* = .206), indicating that participants in both conditions were equally accurate initially. In the first half of trials, participants in both the limited and non-limited groups demonstrated significant improvements in performance (limited: b = .045, SE = .007, p<.001; non-limited: b = .042, SE = .009, p<.001). Thus, the two groups did not differ in learning early in the task (b = −.004, SE = .012, *p* = .777). However, we did find a significant effect of condition on growth across the second half of the trials (b = .010, SE = .005, *p* = .040). Participants led to view willpower as limited did not improve during second half of trials (b = .003, SE = .003 p = .412), whereas participants who led to view willpower as non-limited continued to increase in accuracy (b = .013, SE = .003, p<.001). Only participants in the non-limited willpower condition sustained learning for the entire duration of the task (Figure 1).

These results extend previous work linking motivation to cognitive performance (e.g., [7], [8]) and highlight the interactive nature of motivational and cognitive processes by demonstrating that implicit theories about willpower can affect people’s ability to recruit cognitive resources to sustain learning over time. Whereas previous work assessing the impact of these implicit theories focused on decrements in performance following self-control demands [4], this experiment suggests that people’s beliefs about the nature of willpower can also limit or facilitate the acquisition of a cognitive skill.

The simple nature of the manipulation employed in this study suggests that beliefs about willpower can be easily modified by subtle input, at least in the short-term. This point is especially important given that much recent academic and popular literature claims that human willpower is inherently limited (e.g., [9], [10]). The current findings suggest that disseminating such notions may create self-fulfilling prophecies; leading people to believe that willpower is limited might contribute to decrements in willpower and undermine persistence and learning. Further, the demonstration that implicit theories about willpower can affect performance over a sustained duration stands in contrast to recent work suggesting that these theories only improve performance under relatively mild conditions [11]. The current results instead demonstrate that implicit theories can improve performance even for a very difficult and lengthy task.

The present findings suggest many important directions for future research. For instance, we have shown that theories of willpower influence learning during a task, but we have not determined whether or how long acquired information or skills are retained. Another important open question involves directionality. In particular, we have only demonstrated a relative benefit of priming a non-limited theory of willpower relative to a limited theory. We cannot state whether a non-limited theory improves sustained learning, whether a limited theory undermines sustained learning, or both. Finally, we have taken the approach of priming one theory of willpower, but behaviorally relevant individual differences do exist in beliefs about willpower [4]. It will be important to determine how promoting a belief interacts with preexisting theories of willpower to produce effects on behavior.

The present results are especially meaningful in light of evidence that links training-based improvements in performance on working memory tasks (e.g., a modified n-back task) to increased fluid intelligence [12]. Whereas the working memory training implemented in this previous work was conducted over a series of sessions, the manipulation employed in the present study produced meaningful improvements in performance within a much shorter time frame. A more robust implementation of the intervention employed in the current study may produce effects that compound over time. As people improve on the task, the resulting success may feed back and reinforce the implicit theory that initially gave rise to these advantages. The potential of such a recursive process to expand working memory and fluid intelligence over extended training remains an important topic for future work.

## Supporting Information

Figure S1 **Limited and non-limited questionnaire responses.** The distribution of responses for the limited and non-limited willpower questionnaires. Both groups indicated overall agreement with the questionnaires.
(DOCX)

Text S1 **Limited and non-limited questionnaire items.**
(DOCX)

Text S2 **Growth curve analysis methods.**
(DOCX)
