# Supplementary figures and images for: Theories of Willpower Affect Sustained Learning
Source: PLoS One. 2012 Jun 22;7(6):e38680. doi: 10.1371/journal.pone.0038680 (PMC3382137; doi:10.1371/journal.pone.0038680)

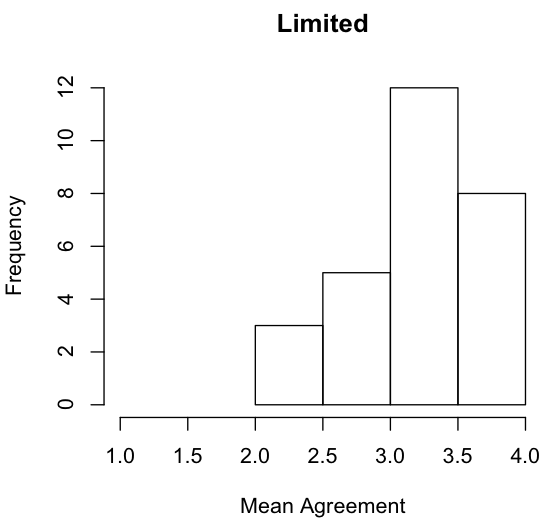


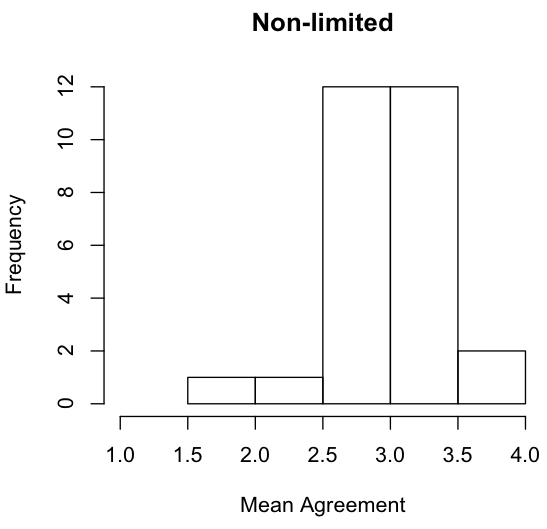

Supplement: Figure S1 — Limited and non-limited questionnaire responses. The distribution of responses for the limited and non-limited willpower questionnaires. Both groups indicated overall agreement with the questionnaires. (DOCX) [file pone.0038680.s001.docx]
