# Supplementary material for: Theories of Willpower Affect Sustained Learning
Source: PLoS One. 2012 Jun 22;7(6):e38680. doi: 10.1371/journal.pone.0038680 (PMC3382137; doi:10.1371/journal.pone.0038680)
Supplement: Text S1 — Limited and non-limited questionnaire items. (DOCX) [file pone.0038680.s002.docx]

***Limited / Non-Limited Questionnaire Items***

Limited:

1. When you think over a matter with great concentration, it can be sometimes tiring.

2. Working on a strenuous mental task can make you feel tired such that you need a break before accomplishing a new task.

3. When you have to do many demanding activities for a while, you eventually get exhausted and less productive.

4. Sometimes, when you completely focus your attention on a demanding mental activity, you feel tired and you need a break sooner or later since your resources have to be refilled.

5. After you have been working on a strenuous mental task for several hours you get fatigued so that you need to rest before taking on the next challenging activity.

6. Strenuous mental activity sometimes exhausts your resources, which you need to refuel afterwards (e.g. through breaks, doing nothing, watching television, eating….).

7. After a strenuous mental activity your energy can be depleted and you sometimes must rest to get it refuelled again.

8. Sometimes, when you have completed a very exhausting mental activity, you have to recover your mental energy again before starting with the same concentration on a new difficult task.

Non-limited:

1. Sometimes, it can be very inspiring to think over a matter with great concentration.

2. It can be energizing to be completely focused on a demanding mental activity, so that you are able to remain concentrated for a while.

3. Sometimes, it is energizing to be fully absorbed with a demanding mental task.

4. It can be energizing to be completely focused on a demanding mental activity, so that you can remain concentrated for a long time.

5. Sometimes, working on a strenuous mental task can make you feel energized for further challenging activities.

6. Sometimes, your mental stamina fuels itself. After a strenuous mental exertion you can continue doing more of it.

7. It is possible to be in such a productive work mode that you don’t need much recreation between different mentally strenuous tasks.

8. Working on a strenuous mental task can activate your mental resources and you become even better at accomplishing subsequent demanding tasks.
