# Supplementary material for: Theories of Willpower Affect Sustained Learning
Source: PLoS One. 2012 Jun 22;7(6):e38680. doi: 10.1371/journal.pone.0038680 (PMC3382137; doi:10.1371/journal.pone.0038680)
Supplement: Text S2 — Growth curve analysis methods. (DOCX) [file pone.0038680.s003.docx]

We calculated a two-part growth model using Mplus 5. To test Slope 1, the slope across the first half of the trials, we set the first four blocks to have weights of 0, 1, 2, 3 and the remaining four blocks to a weight of 3; thus modeling growth for only the first four blocks. To test Slope 2, the slope across the second half of the trials, we set the first four blocks to have a weight of 0 and the remaining four blocks to have weights of 4, 5, 6, and 7. These analyses resulted in an intercept for both groups and two slopes for each group (a slope for each half of the trials). We repeated this analysis using 2, 4, 6, 10, 15, and 20 blocks and found similar results across the analyses.
